# Supplementary material for: Development of a computer-aided design software for the quantitative evaluation of aesthetic damage
Source: PLoS One. 2019 Dec 18;14(12):e0226322. doi: 10.1371/journal.pone.0226322 (PMC6919621; doi:10.1371/journal.pone.0226322)
Supplement: S1 Code site — 1. (ZIP) [file pone.0226322.s001.zip › [EESC_jr][Projeto]Relatorio_07_21/vendors/select2/docs/_includes/options-old/backwards-compatibility.html]

# Backwards compatibility

Select2 offers limited backwards compatibility with the previously 3.5.x
release line, allowing people more efficiently transfer across releases
and get the latest features. For many of the larger changes, such as the
change in how custom data adapters work, compatibility modules were
created that will be used to assist in the upgrade process. It is not
recommended to rely on these compatibility modules, as they will not
always exist, but they make upgrading easier for major changes.

**The compatibility modules are only included in the
full builds of
Select2**. These files end in `.full.js`, and the
compatibility modules are prefixed with `select2/compat`.

## Simplified function for matching data objects

Added in Select2 4.0.0.
This method was added to make upgrading easier from earlier versions of
Select2.

During the Select2 4.0.0 release, the
`matcher` function was changed to allow for more complex
matching of nested objects.

Key
:   `matcher`

Value
:   A function taking a search `term` and the data object
    `text`.

Adapter
:   `oldMatcher`

The custom matcher example provides a
guide for how to use this in your own application. For those upgrading
from older versions of Select2, you just need to wrap your old
`matcher` with this function to maintain compatibility.

## Old initial selections with `initSelection`

Deprecated in Select2 4.0.
This has been replaced by another option and is only available in the
full builds of
Select2.

In the past, Select2 required an option called `initSelection`
that was defined whenever a custom data source was being used, allowing
for the initial selection for the component to be determined. This has
been replaced by the `current` method on the
data adapter.

Key
:   `initSelection`

Value
:   A function taking a `callback`

Adapter
:   `DataAdapter`

Decorator
:   `InitSelection`

## Querying old data with `query`

Deprecated in Select2 4.0.
This has been replaced by another option and is only available in the
full builds of
Select2.

In the past, Select2 supported an option called `query` that
allowed for a custom data source to be used. This option has been replaced
by the `query` method on the
data adapter and takes a very similar set of
parameters.

Key
:   `query`

Value
:   A function taking `params` (including a `callback`)

Adapter
:   `DataAdapter`

Decorator
:   `Query`

## Compatibility with `<input type="text" />`

Deprecated in Select2 4.0.
It is now encouraged to use the `<select>` tag instead.

In past versions of Select2, a `<select>` element could
only be used with a limited subset of options. An
`<input type="hidden" />` was required instead, which did
not allow for a graceful fallback for users who did not have JavaScript
enabled. Select2 now supports the `<select>` element for
all options, so it is no longer required to use `<input />`
elements with Select2.

Adapter
:   `DataAdapter`

Decorator
:   `InputData`
